# Primary Cancer Prevention Through Vaccination: Advances, Challenges, and Global Perspectives

**DOI:** 10.1002/hsr2.72666

**Published:** 2026-06-22

**Authors:** Christian Tague, Johan Domga, Ayesha Junaid, Okhesomi Rejoice Eshemokhai, Josias Kamgang Silatchom, Criss Koba Mjumbe

**Affiliations:** ^1^ Department of Research Medical Research Circle (MedReC) Goma DR Congo; ^2^ Faculté de Médecine Université Libre des Pays des Grands Lacs Goma DR Congo; ^3^ Dow University of Health Sciences Karachi Pakistan; ^4^ Faculty of Clinical Sciences Obafemi Awolowo University Ile‐Ife Osun State Nigeria; ^5^ Faculty of Medicine University of Lubumbashi Lubumbashi DR Congo

**Keywords:** cancer prevention, HBV vaccine, HPV vaccine, oncogenic viruses, vaccination

## Abstract

Oncogenic viral and bacterial infections constitute a significant portion of the global cancer burden, accounting for up to 15% of cases, particularly in low‐ and middle‐income countries. Prophylactic vaccination thus appears to be the most effective tool for preventing these preventable cancers, especially those linked to human papillomavirus (HPV) and hepatitis B virus (HBV). This narrative review synthesizes recent data concerning the impact, advances, limitations, and prospects of available and developing cancer vaccines. Research shows that HPV vaccination significantly reduces the incidence of vaccine‐specific infections, precancerous lesions (CIN2+), and, in the longer term, invasive cancers, with an enhanced effect when vaccination coverage is high and administration is early. Similarly, the universal introduction of the HBV vaccine has led to a remarkable decrease in chronic infections and hepatocellular carcinoma, as evidenced by the successes observed in Taiwan and The Gambia. Despite these advances, challenges remain, including limited access, costs, logistics, sociocultural acceptability, and insufficient vaccination coverage, particularly for the birth dose of HBV and in resource‐limited countries. Furthermore, several prophylactic vaccines against oncogenic agents such as EBV, *H. pylori*, HCV, HTLV‐1, and KSHV are under development, supported by the rise of innovative platforms, notably mRNA.

## 1 Introduction

Oncogenic viral and bacterial infections represent a major public health challenge today, accounting for approximately 15% of cancers worldwide, with a disproportionate burden in low‐ and middle‐income countries. Among these agents, human papillomavirus (HPV) and hepatitis B virus (HBV) remain the most significant, associated respectively with genital and oropharyngeal cancers and hepatocellular carcinoma. Their prevention through vaccination is one of the most effective and efficient strategies for sustainably reducing the incidence and mortality of these preventable tumors [1, 2]. Since the introduction of prophylactic vaccines against HPV and HBV, numerous population studies have demonstrated a major impact on reducing infections, precancerous lesions, and, in some contexts, invasive cancers. These successes give vaccination a central strategic role in the primary prevention of cancer and reinforce the need to accelerate its deployment worldwide [3]. However, despite the availability of safe and effective vaccines, significant disparities persist in access, coverage, and acceptability, hindering the full realization of their preventive potential. This narrative review aims to provide a critical analysis of vaccination as a means of primary cancer prevention, while exploring global advances, challenges, and prospects.

## 2 Methodology

This narrative review was conducted to synthesize current data on primary cancer prevention through vaccination. Scientific articles were identified from the PubMed/MEDLINE, Embase, Cochrane Library, and Google Scholar databases, supplemented by recent institutional reports from the WHO, IARC, GAVI, and UNICEF. Keywords used included: “cancer prevention,” “vaccination,” “HPV vaccine,” “HBV vaccine,” and “oncogenic viruses,” combined using Boolean operators.

Studies were selected without date or language restrictions, but with preference given to publications between 2015 and 2025. Systematic reviews, clinical trials, original articles, observational studies, and official reports on existing (HPV, HBV) or developing (EBV, *H. pylori*, HCV, HTLV‐1, KSHV) cancer vaccines were included. Articles dealing exclusively with therapeutic or experimental vaccines without a direct link to primary prevention were excluded.

The extracted data concerned: the type of vaccine, the population studied, vaccination schedules, the impact on the incidence of infections and cancers, as well as implementation challenges and future prospects. The information was analyzed descriptively and thematically, comparing the results according to geographic regions and economic contexts.

## 3 Vaccination Against Human Papillomavirus (HPV)

### 3.1 Epidemiology of HPV and Associated Cancers

Recent data support a major epidemiological burden of high‐risk HPV in different population segments. For example, the landmark meta‐analysis, which included 93 studies and 29,900 men, shows that the anal prevalence of HPV16 and high‐risk HPV is consistently higher among men who have sex with men (MSM) living with HIV compared to HIV‐negative individuals, with levels ranging from 8.7% for HPV16 and 26.9% for HR‐HPV in HIV‐positive individuals versus 1.8% and 6.9% in HIV‐negative individuals. This risk gradient continues in the age‐related analysis, where an increase in HPV16 is observed with advancing age, followed by an independent link between HIV and the risk of HSIL+ lesions after adjustment [1]. Consistently, in women with current cervical cytological abnormalities, the overall prevalence of HPV reaches 79.1%, with a bimodal dynamic depending on age and an overrepresentation of high‐risk genotypes (notably HPV58, HPV52, HPV53, HPV16, and HPV18), resulting in a highly significant statistical association between HPV infection (whether “any type” or “HR‐HPV”) and abnormal smears [2]. Concurrently, in the field of head and neck oncology, international literature shows that the proportion of oropharyngeal carcinomas attributable to HPV is experiencing a robust temporal increase worldwide: in an aggregation of 9541 specimens from 23 nations, the relative increase in the proportion of HPV‐positive cancers reaches approximately +20% globally, with comparable trends in North America and Europe [3]. Finally, an international time‐trend analysis of over 156,000 oropharyngeal cancers shows a notable rise in incidence in many countries, contrasting with a concomitant decline in pulmonary squamous cell carcinoma and the absence of a net rise in squamous cell carcinoma of the oral cavity, supporting the idea that behavioral population changes (notably the decline in smoking) are gradually shifting ENT epidemiology towards a predominantly HPV‐driven model, with marked cohort effects in the middle and upper age groups [4].

### 3.2 Available Vaccines

a. HPV2 (Cervarix): bivalent (16, 18)
  The availability of the bivalent human papillomavirus vaccine, Cervarix, in Africa appears closely linked to the dynamics of the gradual introduction of national vaccination programs, both in countries with strong logistical capacity and in those supported by international partnerships, and several recent studies have clarified the extent of its distribution. For example, a multicenter survey of 135 otolaryngologists in 19 African countries reported that, among the nine countries that had actually established a national HPV vaccination program, five specifically used Cervarix, illustrating a real, albeit heterogeneous, deployment of the vaccine across the continent; this same study showed that 26 countries had conducted pilot projects or demonstration campaigns, confirming that Cervarix is often included as one of the reference vaccines in the pre‐national phases of operational validation [5]. This availability is particularly well documented in Southern Africa, where South Africa provides a representative example of integration into a large‐scale public program. An economic analysis of the Grade 4 student vaccination strategy demonstrated that the South African government institutionalized a two‐dose Cervarix regimen, supported by robust budget planning from 2014 to 2018, with a projected cost of ZAR 172.7 million for girls' vaccination and ZAR 250 million for a three‐dose regimen. Such investments demonstrate a national commitment to ensuring the accessibility of Cervarix in the public sector and a logistical capacity to support its large‐scale distribution [6].
  The actual availability of Cervarix in Africa is further illustrated by its inclusion in several vaccine feasibility studies conducted in countries heavily affected by cervical cancer, notably Mozambique. A modeling study comparing different vaccines identified Cervarix as a priority strategic option due to its high efficacy and cross‐protection, preventing up to 70% of cervical cancer cases and deaths [5, 7]. Economic projections indicate that a national program based on this vaccine would be feasible in the Mozambican context, with an estimated annual cost of $37 million if funded by Gavi, and between $60 and 81 million without external support. These data confirm that Cervarix is not only accessible but also considered one of the most relevant options for large‐scale implementation in sub‐Saharan Africa [7].
b. HPV4 (Gardasil): quadrivalent (6, 11, 16, 18)
  The availability of the Gardasil−4 vaccine in sub‐Saharan Africa is documented through economic and implementation studies, revealing both sustained national commitments and budgetary challenges related to program sustainability. In Burkina Faso, cost and health impact modeling shows that Gardasil‐4 is one of the vaccines supported by Gavi: the discounted programmatic cost over a decade (2022–2031) is estimated at $35 millions for Gardasil‐4, compared to $24.5 millions for Cecolin and US$37 million for Cervarix, indicating that Gardasil‐4 is financially integrated into national coverage projections [8]. The Burkina Faso study also highlights that, when cross‐protection is taken into account, Gardasil‐4 could prevent up to 48% of cervical cancer cases and deaths in this context, reinforcing the relevance of its use even compared to other vaccines [8, 9]. These projections incorporate phased rollout scenarios, which could be implemented by national authorities in collaboration with partners, demonstrating strategic planning geared towards sustainability [10].
  In Zambia, the assessment of the cost of HPV vaccine delivery shows that Gardasil‐4 is readily available at a price negotiated through Gavi, at $4.60 per dose, a key determinant of the program's viability [9]. Furthermore, the Zambian government's national contribution (co‐financing) is very low ($0.55 per dose), demonstrating that the vast majority of the cost per dose is covered by the Gavi partnership, facilitating access to the vaccine for a large‐scale public program [9, 10]. The same Zambian report indicates that school distribution remains the most cost‐effective strategy: the economic costs per dose are lower in schools ($13.2) compared to health centers, reinforcing the option of school‐based deployment as the preferred mode of vaccination [8, 11]. This strategy not only optimizes logistical resources but also maximizes coverage in a priority age group, thus supporting the actual availability of Gardasil‐4 at the community level.
  Finally, the existence of clinical trials in Africa also demonstrates that Gardasil‐4 is not only used in national programs, but also in operational research: the KENSHE study (Kenya) protocolizes the administration of Gardasil‐4 (types 6/11/16/18) to adolescent girls in a randomized trial, highlighting that this vaccine is approved for routine use in the country and shows the intention to evaluate alternative regimens (such as a single dose), which strengthens the credibility and sustainability of its deployment [10, 11].
c. HPV9 (Gardasil 9): nonavalent (6, 11, 16, 18, 31, 33, 45, 52, 58)
  The availability of Gardasil−9 (a nonavalent vaccine) in Africa is currently largely contingent on economic constraints and vaccination strategy choices. In the case of Burkina Faso, a modeling study by Kiendrébéogo and colleagues estimates that the program using Gardasil‐9 would cost $19.8 millions per year without Gavi support, making this option less viable unless the price per dose is significantly reduced [8, 12]. Furthermore, the same authors show that a single‐dose strategy, if adopted, could substantially reduce the cost of Gardasil‐9 and improve its cost‐effectiveness, suggesting that actual access to the vaccine will depend heavily on the chosen vaccination schedule [8, 13]. These combined analyses indicate that the availability of Gardasil‐9 in Africa remains limited, but has the potential to increase, provided that economic and market barriers are overcome through price negotiations, external financing, and adaptations to vaccination strategies.

### 3.3 Impact Data on HPV Incidence and Precancerous Lesions

The implementation of human papillomavirus (HPV) vaccination programs has led, at the population level, to marked reductions in the prevalence of infections by vaccine types and to herd effects in sexes and cohorts not targeted by initial vaccination. This has been demonstrated by an international meta‐analysis documenting a significant decrease in HPV 16/18 infections and a reduction in the incidence of precancerous lesions (CIN2+) in young women in vaccinated cohorts, providing evidence that well‐covered programs rapidly alter the biological burden of HPV at the population level [11, 14].

Several reviews and meta‐analyses focused on actual efficacy show substantial reductions in the incidence of CIN2+ in the age groups most exposed to vaccination, with particularly marked decreases in young vaccinated women (adolescents and young adults) and convergent trends between studies from countries with high coverage; these analyses also highlight that the magnitude of the reduction is strongly dependent on the age at vaccination, with administration before sexual exposure conferring the greatest benefits, and report weighted reductions in CIN2+ consistent between clinical trials and observational field studies [12, 13].

From an epidemiological perspective on cancers, large‐scale cohort data show that over time, quadrivalent and bivalent vaccination is associated with a decrease in the incidence of precancerous lesions and, in the longer term, a decrease in the incidence of invasive cancer associated with the vaccine types; A Swedish population study and several national series have demonstrated an association between vaccination and a reduced risk of invasive cervical cancer, confirming that primary prevention through vaccination leads to measurable effects on tumor morbidity when coverage is sufficient and implementation extends over several years [14, 15, 16, 17, 18].

In summary, the body of published evidence indicates that HPV vaccination results in a robust and reproducible decrease in the incidence of vaccine‐targeted HPV infections and precancerous cervical lesions (CIN2+). These effects are maximized when vaccination is administered before exposure (adolescence), when coverage is high, and when programs are accompanied by appropriate screening. These findings are corroborated by meta‐analyses and long‐term national series and now constitute the central argument for expanding vaccination programs and maintaining high coverage.

### 3.4 Implementation Challenges

The implementation of human papillomavirus (HPV) vaccination programs faces several structural and contextual obstacles that directly influence access to and coverage of the vaccine in low‐ and middle‐income countries, particularly in sub‐Saharan Africa. Supply constraints, logistical costs, and dependence on international partners such as Gavi are major factors limiting the effective availability of doses in some regions, despite the existence of formalized national programs [15]. In this context, countries often have to prioritize specific cohorts (girls aged 9–14) and use school‐based vaccination strategies to maximize coverage while circumventing supply and funding limitations [19, 20].

The acceptability of vaccination represents a second major challenge. Several studies have documented that social, cultural, and religious perceptions can influence the decisions of parents and adolescent girls to get vaccinated, even when the vaccine is available and free. Lack of awareness of the benefits, fears of side effects, and the stigma associated with sexually transmitted infections are common barriers that require targeted community awareness and education interventions to improve uptake [16, 21]. These factors affecting acceptability are often correlated with the inclusion of local communities in planning and proactive communication from health authorities regarding vaccine safety and efficacy.

Finally, vaccination coverage is highly dependent on a combination of access and acceptability. In countries where vaccination is integrated into the school system, initial coverage can reach high levels (> 80%), but vaccinating girls outside of school remains a persistent problem, reducing the overall effectiveness of the program at the national level [17]. Furthermore, the frequency of two‐ or three‐dose schedules, the intermittent availability of vaccines, and logistical challenges (transport, temperature‐controlled storage, cohort monitoring) complicate achieving complete and uniform coverage, particularly in rural or isolated areas.

## 4 Hepatitis B (HBV) Vaccination

### 4.1 Link Between Chronic HBV and Hepatocellular Carcinoma (HCC)

Chronic hepatitis B virus (HBV) infection is a leading cause of hepatocellular carcinoma (HCC), causing more than 820,000 deaths annually worldwide [19, 20]. HBV is responsible for 50%–80% of HCC cases, especially in sub‐Saharan Africa and East Asia [20]. The risk of HCC is significantly high in individuals who are infected perinatally or in early childhood, because chronic infection is more likely to develop and persist in them [10, 19, 20]. The link between HBV and HCC emphasizes the importance of HBV vaccination as a primary strategy to prevent cancer [19, 20, 21].

### 4.2 HBV Vaccination: Types, Schedules, Integration Into EPI Programs

HBV vaccines are recombinant in nature, safe, and highly effective. The routine schedule consists of three doses, the first dose is given within 24 h of birth, followed by two or three additional doses [20, 22]. The World Health Organization (WHO) has recommended universal infant vaccination integrating with their national immunization programs (EPI) [23]. Newer vaccines, such as HEPLISAV‐B, is a two‐dose vaccine for adults, which helps in improving compliance [20]. By 2019, global coverage for the three‐dose vaccination reached 85%, but birth dose coverage still remained lower at around 46% [20].

### 4.3 Demonstrated Impact on the Decline of CHC

Universal HBV vaccination had a remarkable reduction in chronic HBV infection and HCC incidence. Taiwan provides one of the strongest examples: after introducing universal infant vaccination in 1984, it resulted in a drop in HBsAg prevalence from 9.78% to 0.64% [24, 25]. Similar successes have been observed in The Gambia and China, where vaccination programs have significantly reduced HBV infection and liver cancer rates [26]. It is estimated that if timely birth‐dose vaccination reached 90% across low‐ and middle‐income countries, more than 700,000 deaths could be prevented by 2030 [27].

### 4.4 Challenges in PRFIs (Birth Dose, Incomplete Coverage)

Despite the effectiveness of the HBV vaccine, challenges still persist, especially in perinatal risk factor interventions (PRFIs). Global birth dose coverage remains below the target, particularly in Africa and Southeast Asia, due to barriers such as home births, cold chain infrastructure limitations for vaccine storage, vaccine hesitancy, and supply issues [19, 20, 27, 28]. However, introduction of the vaccine and administration at birth resulted in a significant reduction in mother‐to‐child transmission, chronic HBV infection, and HCC [29]. Addressing these gaps is essential in order to achieve elimination targets.

### 4.5 WHO Recommendations and Eradication Prospects

The WHO aims for 90% HBV vaccination coverage and a 65% reduction in HBV‐related deaths by 2030 [30]. These goals can be achieved by improving birth dose coverage, integrating vaccination with maternal screening and antiviral therapy, and strengthening health systems [19, 20]. While elimination is possible, sustained political commitment, funding, and innovative delivery strategies are essential for vaccine coverage and reducing HBV‐related deaths [19, 20].

## 5 Cancer Vaccines in Development or Under Investigation

### 5.1 Epstein‐Barr Virus (EBV) and Lymphomas/Hodgkin

EBV is associated with several cancers, including Hodgkin lymphoma and nasopharyngeal carcinoma [31]. Recent vaccine development includes subunit, viral vector, nanoparticle, and mRNA‐based vaccines, which target key viral antigens such as gp350, gp42, gH/gL, and latency proteins [31]. Although preclinical and early clinical trials have shown success, further research is needed to prove the efficacy in cancer prevention. However, no vaccine is available yet.

### 5.2 *Helicobacter pylori* and Gastric Adenocarcinoma

*H. pylori* is a major cause of gastric adenocarcinoma. Vaccine development is still under progress, with several trials in preclinical and early clinical stages. Challenges include antigenic diversity and the need for strong mucosal immunity for vaccine development. No vaccine is yet available for public use [32].

### 5.3 Hepatitis C Virus (HCV) and HCC

HCV is a significant cause of HCC [21]. Vaccine development is complicated by the virus's genetic diversity and variability. Although several ongoing clinical trials, no effective prophylactic HCV vaccine is currently available [33].

### 5.4 HTLV‐1 Virus, Kaposi‐Associated Herpesvirus (KSHV)

HTLV‐1 is linked to adult T‐cell leukemia/lymphoma, and KSHV causes Kaposi sarcoma [34]. Both of them are being worked on for vaccine development, but progress is still limited to preclinical studies and early‐phase trials [35].

### 5.5 Innovative Platforms: mRNA, Recombinant Proteins, Therapeutic Vaccines

The mRNA vaccines being successful for COVID‐19 have also resulted in increased interest in mRNA‐based cancer vaccines. These vaccines have rapid development, high potency, and adaptability. Clinical trials are under progress for mRNA vaccines which target melanoma, HPV‐related cancers, and other solid tumors [36]. Recombinant protein and peptide vaccines, as well as therapeutic vaccines, are also being explored for various oncogenic viruses [17, 37].

## 6 Perspectives and Recommendations

The next steps in the fight against cancers linked to viral infections rely on several priority areas. The universal introduction of vaccines against human papillomavirus (HPV) and hepatitis B virus (HBV) must be accelerated, particularly in high‐incidence countries where vaccination coverage remains insufficient. The development of new prophylactic vaccines, aiming for broader protection or increased efficacy in high‐risk groups, is also an area of research that needs strengthening.

The systematic integration of vaccination into comprehensive cancer control strategies (national cancer control plans, universal health coverage programs) is a key lever for improving access to and adherence to primary prevention. Furthermore, promoting local vaccine production capacity, combined with cost‐reduction mechanisms, could facilitate sustainable implementation in low‐ and middle‐income countries.

Finally, improving post‐vaccination surveillance systems, including pharmacovigilance and strengthening cancer registries, remains essential to measure the true impact of vaccination programs, identify disparities, and guide public health policies.

## 7 Conclusion

Vaccination is now one of the most effective, safe, and cost‐efficient tools for primary cancer prevention. The results obtained with HPV and HBV vaccines, in terms of both reducing incidence and mortality, exemplify the potential of vaccination strategies in the field of oncology. These successes should encourage a broader commitment to other infectious agents involved in carcinogenesis, in order to expand the scope of vaccine‐based prevention.

However, the growth of preventive oncology will depend on several conditions: sustained political support, long‐term funding, and an innovative capacity geared towards the development of new prophylactic or therapeutic vaccines. In this context, integrating vaccination into national cancer control policies, strengthening epidemiological surveillance infrastructure, and ensuring equitable access to vaccines are crucial challenges. Together, these elements will determine the capacity of health systems to sustainably reduce the global burden of preventable cancers.

## Author Contributions

**Christian Tague:** conceptualization, investigation, writing – original draft, writing – review and editing, validation, methodology, project administration, and supervision. **Johan Domga:** conceptualization, writing – original draft, investigation, writing – review and editing, and validation. **Ayesha Junaid:** writing – original draft, investigation, and validation. **Okhesomi Rejoice Eshemokhai:** writing – original draft, and investigation, validation. **Josias Kamgang Silatchom:** investigation, writing – original draft, and validation. **Criss Koba Mjumbe:** investigation, writing – original draft, validation, project administration, and supervision.

## Funding

The authors have nothing to report.

## Ethics Statement

The authors have nothing to report.

## Conflicts of Interest

The authors declare no conflicts of interest.

## Data Availability

Data sharing is not applicable to this article as no data sets were generated or analyzed during the current study.
